# The Influence of Food Colors on Emotional Perception and Consumer Acceptance: A Sensory and Emotional Profiling Approach in Gastronomy

**DOI:** 10.3390/foods14223818

**Published:** 2025-11-07

**Authors:** Jarbas Silva, Francisca Elisângela Lima, Clarisse Souza, Bruno Moreira-Leite, Paulo Sousa

**Affiliations:** 1 Institute of Culture and Art, Federal University of Ceara, Av. Mister Hull, s/n-Campus do Pici, Fortaleza 60440-554, Brazil; felisangela@ufc.br; 2 Department of Food Engineering, Federal University of Ceará, Av. Mister Hull, s/n-Campus do Pici, Fortaleza 60440-554, Brazil; 3 Department of Chemistry, NOVA School of Science and Technology, NOVA University of Lisboa, Quinta da Torre, 2829-516 Caparica, Portugal

**Keywords:** multisensory perception, consumer behavior, emotional profiling, plating design, crossmodal correspondences, food acceptance, gastronomy

## Abstract

Food color is a powerful determinant of consumer perception, influencing emotions, taste expectations, and hedonic responses. This study investigated how red, yellow, and blue plating colors affect emotional responses, acceptance, and taste associations. Emotional descriptors were defined through two focus groups (n = 17) and validated in a consumer study with 295 participants (63.4% female, 35.3% male). Three color-dominant samples were evaluated online using the Check-All-That-Apply (CATA), Rate-All-That-Apply (RATA), and a nine-point hedonic scale. The red sample achieved the highest acceptance (7.27), followed by blue (7.03) and yellow (6.82) (*p* < 0.05). Red was strongly associated with positive RATA terms such as *pleasant* (3.90), *with pleasure* (2.95), and *satisfied*, while blue elicited negative responses, including *disgusted* (72%) and *no appetite* (74%). Pearson correlations confirmed *pleasant* (r = 0.70, *p* < 0.001) and *with pleasure* (r = 0.58, *p* < 0.001) as key acceptance drivers, whereas *disgusted* (r = −0.29, *p* < 0.001) acted as a rejection cue. Correspondence analysis explained 68% of the variance, and Partial Least Squares Regression highlighted *pleasant* (VIP = 1.86) as the strongest predictor of liking. Tableware (≥4.25) and plating arrangement (≥4.31) also significantly shaped emotional perception. These results demonstrate that plating colors critically influence consumer emotions and acceptance, offering practical insights for multisensory gastronomy and food design. Overall, the study shows that plating color can be strategically leveraged in gastronomy and product development to enhance consumer emotions and acceptance, providing valuable guidance for multisensory food design. Although conducted using photographic stimuli and limited to Brazilian consumers, the study provides valuable insights into how plating color influences emotional and hedonic responses. These findings can support both academic research and professional practice, guiding chefs and food designers in developing multisensory gastronomic experiences.

## 1. Introduction

Color is one of the primary attributes and criteria for selecting and accepting gastronomic products and preparations, whether of plant or animal origin. It is inherent in food pigments, which can be derived from various sources, including microorganisms, animals, insects, and, most abundantly, plants. These pigments are classified into distinct groups, such as anthocyanins (responsible for blue, purple, and red hues), betalains (yellow to red, found mainly in beets and pitaya), carotenoids (orange, red, and yellow shades), and chlorophylls (responsible for the green color of vegetables) [1].

The perception of color is multifaceted, as different fields of study—physics, psychology, vision science, and the arts—have proposed theories to explain it. From a physical perspective, color is related to the behavior of light wavelengths. White light, being polychromatic, comprises all wavelengths within the visible spectrum. The human eye contains optical components such as the cornea, iris, and retina, which work together with a neural network to generate visual perception. Equipped with S, M, and L cones sensitive to short, medium, and long wavelengths, the human eye perceives primary chromatic signals (blue, green, and red). Other colors, such as yellow, arise from combined cone stimulation, while white results from the balanced activation of all cones and black from the absence of light. The sensory process of color perception begins with the stimulation of light receptors, where stimuli are converted into electrical signals. These signals are then processed in the visual cortex via the optic nerves, resulting in the perception of color [2,3].

Beyond these physical and biological explanations, traditional color theory provides an alternative perspective. This approach emphasizes the color of pigments and recognizes the complexity of color, including its physical, perceptual, and psychological dimensions. It explores color through ontological and epistemological lenses, highlighting its applications in diverse contexts while distinguishing itself from theories of color vision or color science [4].

In gastronomy, color plays a critical role in the visual aesthetics of culinary preparations. Through sight, consumers assess the composition and arrangement of food on a plate, underscoring the significance of visual appeal—often described as “eating with the eyes” [5,6]. According to Schifferstein et al. [6], plating represents an artistic arrangement of food elements that creates the consumer’s first interaction with the dish. Although empirical studies on optimal presentation methods remain scarce, visual attributes such as color, shape, texture, gloss, and size significantly influence attractiveness, sensory perception, and acceptance [7]. Furthermore, consumers rely heavily on color to assess the safety, quality, and ripeness of vegetables, drawing inferences about sensory attributes such as taste and texture [8].

Colors affect not only perception but also psychological and emotional responses. Psychological theories suggest that color reliably evokes emotions and can distinguish between them. This relationship is particularly evident in food-related contexts, where color and emotion shape consumption behavior, mood, and the affective responses elicited by food. Since emotions are often expressed spontaneously during food consumption, sensory researchers have shown increasing interest in measuring these responses [9,10,11].

Although the role of color in food perception has been widely examined, most studies have concentrated on conventional color–flavor correspondences—such as the association of red with sweetness or yellow with sourness—and on marketing or packaging contexts rather than plated dishes [6,12,13,14]. Empirical evidence on how plating colors evoke emotional responses remains limited, particularly when less typical hues such as blue are considered. Because natural blue foods are rare, their use in gastronomy often induces perceptions of strangeness or rejection, contrasting with the generally positive symbolism of blue in other contexts [15,16,17]. This gap underscores the need to investigate how non-traditional plating colors influence consumer emotions and acceptance. By integrating hedonic evaluation with Check-All-That-Apply (CATA) and Rate-All-That-Apply (RATA) emotional profiling, the present study advances understanding of the sensory–emotional effects of plating colors. It contributes to a broader perspective on crossmodal correspondences in multisensory gastronomy.

In addition to the influence of color, the form and arrangement of food elements on the plate also play a critical role in shaping consumer responses. Previous research confirmed that the spatial organization of components directly affects hedonic evaluation. Michel et al. [18] demonstrated that both the shape and arrangement of elements in a dish can significantly alter perceptions of attractiveness, taste, and overall liking, emphasizing that plating design is not merely an aesthetic choice but a multisensory cue that contributes to consumers’ sensory and emotional experiences.

Three primary hues—red, yellow, and blue—were selected to represent the foundational dimensions of color theory. This choice allowed for a controlled comparison among colors with distinct psychological connotations, avoiding overlap and perceptual ambiguity that could arise from testing multiple intermediate tones. Future research may expand this design by including secondary or desaturated hues to explore the full color spectrum in gastronomy.

Therefore, this study aimed to investigate how plating colors influence consumer emotional responses, hedonic acceptance, and associations with basic tastes and sensations. Specifically, we hypothesized that plating colors would significantly affect both emotional perception and consumer acceptance, with red eliciting predominantly positive emotions, blue evoking negative ones, and yellow producing intermediate responses.

In addition, this research contributes to the emerging field of digital sensory evaluation, in which photographs and online methods are increasingly used to assess visual-emotional responses. Recent advances in this area have demonstrated that digital stimuli can reliably capture perceptual and affective reactions, expanding the methodological possibilities for multisensory food research [19,20].

## 2. Materials and Methods

To investigate the emotional effects associated with the perception of food colors in plating, the Check-All-That-Apply (CATA), Rate-All-That-Apply (RATA), and Hedonic Scale tests were employed to evaluate consumer acceptance of three samples within the framework of sensory analysis.

This study was conducted with untrained consumers rather than expert sensory panelists. Such an approach aligns with best practices in sensory and consumer science, as the primary objective was to assess hedonic acceptance and emotional responses, which representative consumers of the target population most appropriately evaluate.

### 2.1. Sample Selection

The use of pre-existing plated dishes, sourced from professional gastronomy photography, ensured that the visual stimuli represented realistic and aesthetically consistent food presentations. Preparing new dishes for this exploratory phase might have introduced variability in plating quality and lighting conditions, compromising visual standardization. The selected images, captured by professional food photographers, provided high fidelity in color and texture representation, thereby supporting the study’s focus on visual perception rather than gustatory evaluation.

Samples were selected by prioritizing preparations that incorporated colors associated with the primary hues of traditional color theory—namely, dishes prepared with red, yellow, and blue tones. Accordingly, three plating arrangements were chosen, as shown in Table 1. All samples and their specifications were sourced from professional profiles specializing in food and gastronomy photography on Instagram.

### 2.2. Focus Group

To adapt an analysis form for the study, the focus group methodology was employed to discuss the most appropriate ways to conduct the analysis and to define the emotional terms used in the CATA and RATA tests. These terms were initially preselected based on the EsSense Profile^®^ [24], the emotional lexicon for coffee tasting developed by Souza et al. [25], and the emotional lexicon for beer consumption presented by Chaya et al. [26]. Furthermore, the adaptation and suitability of these terms, as well as the need for additional inclusions, were evaluated in two distinct focus groups, as proposed by Minim [27].

Focus group sessions were conducted remotely via the Google Meet platform in July 2021, in compliance with restrictions imposed by the COVID-19 pandemic. The procedure followed established protocols and best practices for conducting at-home sensory evaluations, an approach that has been previously validated and successfully applied in sensory research, including focus group methodologies [28,29].

The first group comprised 10 participants (five female and five male) aged 18–50 years, while the second group included seven participants (three female and four male) aged 18–35 years. Each session lasted approximately 90 min and was held between 5:00 p.m. and 7:00 p.m. Participants were students enrolled in the Bachelor’s degree program in Gastronomy at the Federal University of Ceará. Sessions began with an introduction to the study, providing context on emotional sensory analysis, the samples, and the platings, followed by the presentation of the preliminary list of emotional terms.

The focus groups were composed of gastronomy students due to their familiarity with visual and sensory aspects of food, which facilitated the refinement of emotional descriptors. This selection ensured informed discussions while maintaining the exploratory nature of the study.

The groups were guided to evaluate the suitability of the terms for the samples, identifying the emotions elicited by observing the colors of the food components in the plating arrangement. Each previously selected term was discussed in detail, with participants deciding whether it should be retained, modified, or removed, and whether additional terms should be added.

The three sample options were then presented, and participants were instructed to observe the plating for a few minutes, identify the most prominent color and other colors present in the sample, and describe their emotional responses to each sample.

The data collected from these sessions were compiled into reports to enhance the list of terms for the CATA and RATA tests and to support the broader research findings. The following terms were suggested for inclusion: with appetite, comfortable, instigated, and motivated.

During the analysis, the groups raised four key questions relevant to the study:

1. Does the plate used in the sample affect the perception of emotions?
2. To what extent does the individual like the color in the sample?
3. Is the emotional perception based on the main color or the combination of all colors?
4. Do the shape and arrangement of the elements influence the perception of emotions?

The qualitative reports generated from the two focus group sessions provided valuable insights for defining the terms in the CATA and RATA tests and for supporting the discussion of the results.

After the focus group sessions, the refined list of emotional terms was reviewed by three experts in sensory and consumer science, each with experience in emotional sensory analysis. These specialists independently assessed the adequacy, clarity, and relevance of the terms for use in consumer testing. Their feedback was incorporated to ensure that the final lexicon was valid, comprehensive, and aligned with current practices in affective sensory evaluation.

### 2.3. Sensory Analysis

To collect data, a questionnaire was developed using the Google Forms platform and distributed online through social media channels, including WhatsApp and Instagram (widely used in Brazil), in July 2021. Employing the snowball sampling technique, participants were encouraged to share the survey within their networks to increase the response rate and diversity of participants. No compensation or reward was offered, and participants were fully informed about the study’s objectives through a consent form presented at the beginning of the questionnaire.

A total of 318 participants were invited to participate in the analyses in Brazil. However, due to incomplete questionnaires and the exclusion of 18 colorblind individuals, 295 responses were retained. Participants with color vision deficiencies were excluded to avoid bias in the evaluation of plating colors, as color-deficient individuals perceive chromatic stimuli differently, which could compromise the reliability of sensory and emotional responses.

Among the participants, 63.4% were female (n = 187), 35.3% male (n = 104), 0.3% non-binary (n = 1), and 1% preferred not to disclose their gender (n = 3). Regarding involvement with gastronomy, 50.2% (n = 148) of participants reported no affiliation, while the remaining 49.8% were distributed as follows: 16.6% (n = 49) professionals, 14.2% (n = 42) gastronomy teachers, and 19% (n = 56) gastronomy students.

The recruited participants represented a convenience sample of Brazilian consumers, which is adequate for consumer affective testing but may limit generalization to other populations. Participants were naïve consumers, not trained sensory panelists, in accordance with best practices for acceptance and emotion-based studies.

The questionnaire was structured into 27 sections, and its design was based on observations from the focus group to ensure comprehensive sensory and emotional coverage. For each sample, the questions were organized into three blocks: the first assessed sample acceptance using a nine-point hedonic scale, a list of emotional terms, and a preference measure; the second evaluated the impact of tableware on emotional perception; and the third examined how the shape and arrangement of elements in the plating influenced emotional responses. The form advanced only when all mandatory questions had been answered.

The initial section presented the consent form and a screening question to identify color blindness, which was an exclusion criterion for the study. Participants who indicated that they were color blind were instructed to exit the survey. The second section asked participants to specify whether they were completing the survey on a mobile device or a computer. Subsequent sections included the CATA/RATA test and a nine-point hedonic scale for overall sample evaluation (1 = dislike extremely, 9 = like extremely).

The CATA and RATA questionnaires were based on validated emotional lexicons adapted from the l EsSense Profile ^®^ [24], the coffee emotion lexicon developed by Souza et al. [25], and the beer emotion lexicon by Chaya et al. [26]. The CATA section asked participants to select all emotional terms that applied to each sample, whereas the RATA section required them to rate the intensity of each selected emotion on a five-point scale (1 = a little, 5 = a lot). This combination enabled both qualitative identification and quantitative assessment of emotional responses, consistent with previous sensory emotion research.

In the first evaluation section, participants were presented with the sample, displayed as a photograph, and asked to identify the most prominent or dominant color of the food, as well as any additional colors perceived. Next, participants were asked how much they liked the overall appearance of the sample. Afterwards, they were presented with the emotional terms defined during the focus groups. Initially, participants selected the terms they associated with the sample, and then they were directed to a follow-up section with a five-point intensity scale to rate the intensity of each term (1 = a little, 5 = a lot).

The second block included a question regarding the influence of the plate on emotional perception. If participants responded positively, they proceeded to a section containing two additional questions: one assessing the perceived degree of influence on a five-point scale and another determining whether the influence was positive or negative. Participants who responded negatively or answered “I don’t know” were directed to the third block.

In the final block, participants were asked whether the shape and arrangement of the elements in the plating (i.e., the presentation of food on the plate) influenced their perception of emotions. If they responded positively, they proceeded to rate the intensity of this influence on a five-point scale. If they responded negatively, they were directed to indicate whether their emotional perception was based solely on the main color or on a combination of colors in the sample. After responding, participants proceeded to the next sample, repeating the same sequence of questions.

After evaluating all three samples, participants completed a section with checkboxes to identify the basic tastes and oral sensations associated with each sample. Eight options were provided: sweet, salty, bitter, sour, umami, fatty, astringent, and spicy. The samples were presented in columns, allowing participants to associate the tastes and sensations with each one.

The penultimate section consisted of a demographic and socioeconomic questionnaire, including questions on gender, age group, marital status, education level, and family income. Participants were also asked about their involvement in gastronomy, with four response options: I am a student, I am a teacher, I am a professional in the field, and I am not involved. The final section thanked participants, provided the authors’ contact information, and included links to the Instagram profiles from which the sample images were obtained.

To reduce bias, samples were presented using standardized photographs without any identifying information beyond the plating itself. All images were coded and displayed under the same resolution and background to avoid unintentional visual cues that could influence responses.

To minimize confounding effects related to shape or arrangement, all images were pre-screened to ensure similar composition, lighting, and balance between plate and background. Moreover, participants were asked specific questions regarding the perceived influence of tableware and arrangement, which were analyzed separately, allowing for an explicit assessment of these contextual factors.

The use of high-quality photographs as visual stimuli was a deliberate methodological choice, aligning with validated approaches in sensory and consumer science during the COVID-19 pandemic. Recent studies have confirmed that online evaluations using standardized images can reliably capture emotional and hedonic responses to visual food cues, particularly when investigating perceptual phenomena such as color [19,20,30]. While direct evaluations could offer richer multisensory data, this study specifically aimed to isolate the visual dimension of color perception. Therefore, the use of photographs was not a limitation, but a controlled methodological condition designed to ensure consistency and comparability across stimuli.

### 2.4. Ethics Approval

This study was conducted in accordance with the ethical principles of the Declaration of Helsinki. All participants provided informed consent prior to participation. The research protocol was approved by the Research Ethics Committee of the Federal University of Ceará (protocol number 5.727.033/2021).

### 2.5. Statistical Analysis

All statistical analyses were performed using XLSTAT, version 2025.1 (Addinsoft, Paris, France). Hedonic scores for overall liking and additional structured questions were analyzed by one-way analysis of variance (ANOVA), followed by Tukey’s post hoc test to identify differences among samples. When assumptions of normality and homogeneity were not met, the Friedman test was applied as a non-parametric alternative, followed by Bonferroni-adjusted pairwise comparisons.

Check-All-That-Apply (CATA) responses were analyzed using Cochran’s Q test to detect significant differences among samples, with post hoc pairwise comparisons carried out using McNemar tests. For Rate-All-That-Apply (RATA) data, mean ANOVA and Tukey’s post hoc test were used to analyze intensity scores.

Pearson’s correlation coefficients were calculated to assess associations between hedonic acceptance and RATA emotional descriptors. A heatmap was generated to visualize correlation patterns. In addition, Correspondence Analysis (CA) was applied to CATA data to graphically explore sample–attribute relationships.

Prior to applying parametric tests, data were checked for normality using the Shapiro–Wilk test and for homogeneity of variances using Levene’s test. For the PLSR model, cross-validation was performed to ensure robustness, and the explained variance was reported to evaluate model fit.

Partial Least Squares Regression (PLSR) was used to model the relationship between RATA terms and overall liking. Regression coefficients were extracted to determine the direction and magnitude of associations, and Variable Importance in Projection (VIP) values were computed to identify the main predictors (VIP > 1).

All tests adopted a significance level of *p* < 0.05. Results are reported as means ± standard deviations or standard errors, as indicated in tables and figures.

## 3. Results and Discussion

### 3.1. Focus Group for Defining CATA and RATA Terms

During the sessions, several terms were reviewed to assess their suitability for describing emotions triggered by observing the colors of the plated food. Terms such as amusing, bored, authentic, and classic were excluded due to their lack of relevance to the analysis. Synonymous terms, including disappointed and disillusioned, were consolidated into a single descriptor: dissatisfied. Furthermore, terms related to the characteristics of the prepared food rather than to emotional perception, such as appetizing and freshness, were reconsidered and adjusted. For instance, appetizing was replaced with appetite, while fresh was replaced with refreshed.

New terms such as instigated and comfortable were proposed during the analysis of the samples. Additionally, participants raised four critical questions: the influence of plates on emotional perception, the role of individual colors versus color combinations, the preference for the predominant color, and the impact of the shape and arrangement of elements on emotional perception.

### 3.2. Check-All-That-Apply Analysis

The CATA methodology provided a comprehensive overview of how consumers associated emotional and sensory attributes with the three color-based samples. As shown in Table 2, the McNemar test with Bonferroni correction revealed significant differences in the frequencies of attribute selection, underscoring the impact of food color on consumer perception.

Sample 1 (Yellow), containing mainly yellow elements, displayed the highest frequencies for curious (92%), refreshed (82%), and instigated (82%), confirming its strong association with stimulation and novelty. It also reached elevated values for strange (81%) and thoughtful (81%), showing a dual character that combined positive feelings of curiosity and refreshment with more ambivalent perceptions such as strangeness. Yellow is associated with joy and happiness, as well as optimism, friendliness, and extroversion. It is also linked to acidic sensations in the mouth, which can enhance the perception of refreshment [2,31,32]. However, some negative associations remain. When combined with other colors, feelings such as envy, jealousy, and insecurity may appear. Yellow is also linked to certain pathological conditions, such as jaundice, and to the concept of death [2,33]. Considering the literature, the yellow sample is coherent, reinforcing that the effects of yellow can also be observed in food colors and plating.

Sample 2 (Red), containing mostly red elements, was predominantly associated with positive emotional terms. It showed the highest frequencies for pleasant (95%), with pleasure (85%), intense (83%), and satisfied (79%), reinforcing red as a color strongly linked to attraction and passion, in addition to its association with happiness [2]. Although red has been linked to negative feelings in previous studies—such as guilt when choosing a product, greater susceptibility to errors, impaired cognitive performance in learning situations, and motivation to avoid threats or danger [34,35,36,37]—only positive associations were observed in this study. This suggests that the presence of red elements in food and plating can predominantly evoke positive feelings.

Sample 3 (Blue), containing mostly blue elements, was associated with emotions opposite to those observed in the red sample, particularly negative feelings such as disgusted (72%), no appetite (74%), and strangeness (80%), indicating clear rejection tendencies. The literature provides various meanings for blue and its influence on emotions. Blue is often associated with positive sensations such as sympathy, harmony, friendship, trust, greatness, infinity, concentration, tranquility, peace, and intelligence [2,29,38,39]. However, although blue generally evokes positive emotions, negative associations are more common when it appears in foods. According to [40], blue-colored foods often create a sense of strangeness, giving the impression of artificiality due to synthetic colorings, which can interfere with flavor perception. This is largely because natural blue foods are rare, making it difficult to associate the color with a positive sensory experience. Although some blue foods, such as sweets and cakes, are considered acceptable, consumers still tend to link them with artificially colored products.

Cultural context likely played a crucial role in shaping the observed responses. In Brazilian culture, warm colors such as red and yellow are often associated with vitality, festivity, and abundance, whereas blue tends to evoke distance or coldness. Such symbolic associations differ across regions and could explain the observed preference patterns. Future research should include participants from diverse cultural backgrounds to test the stability of these associations and to assess whether color–emotion correspondences hold across cultural contexts.

Overall, the results partially confirmed the initial hypothesis. As expected, red was associated with predominantly positive emotions and higher acceptance scores, while blue elicited negative emotional responses. However, yellow showed mixed results, with both refreshing and ambivalent associations, indicating that its emotional interpretation may depend on contextual or cultural factors. These findings highlight the complexity of color–emotion relationships and suggest that some correspondences may not be universal but instead culturally mediated.

These results are consistent with previous studies demonstrating that warm colors, such as red and yellow, enhance perceived pleasure and stimulate appetite, whereas cool colors, such as blue, often suppress appetite or evoke aversion.

Color psychology suggests that red attracts attention and is associated with foods perceived as sweeter and more intense [12,41]. Red has been the beverage color most consistently chosen as the sweetest in large-scale studies [42]. Yellow and orange convey joy and optimism and are frequently used in marketing to create a sense of urgency and energy, as well as to stimulate the palate [12]. In contrast, blue is a rare hue in natural foods [12], which contributes to consumer aversion [43,44]. For instance, viewing blue soup has been shown to reduce willingness to eat and increase feelings of anxiety [45].

However, the magnitude and meaning of these effects vary substantially across food categories and, crucially, depend on cultural context and previous experiences [46]. Color–emotion associations are highly context-sensitive, suggesting that familiarity, cultural symbolism, and learned experiences modulate the direction and strength of emotional responses. Evidence shows that although blue is one of the most preferred colors in non-food contexts, such as art [47], its preference drops dramatically when associated with foods or beverages [44]. This contextual dependence is evident in cross-cultural studies, such as that of [48], which found differing flavor expectations (mint versus raspberry) for a blue drink among consumers from Taiwan and the United Kingdom. The cultural aversion to blue has even been intentionally explored by figures such as the futurist Marinetti (1932/2014) [49] and filmmaker Alfred Hitchcock [50] to disorient or unsettle diners.

Taken together, these findings reinforce the importance of visual cues in shaping emotional and hedonic responses to food. The significant differences revealed by the CATA analysis confirm that consumers rely heavily on color as a determinant of both acceptance and rejection. In particular, the consistent preference for red across positive descriptors supports its role as a powerful driver of food attractiveness, while the negative associations with blue highlight cultural and perceptual boundaries in food color design [32,51].

### 3.3. Rate-All-That-Apply Analysis

The RATA methodology revealed clear differences in emotional and sensory perceptions across the three color-based samples (yellow, red, and blue) (Table 3).

The Red Sample consistently achieved the highest mean scores for positive attributes such as pleasant (3.90) and with pleasure (2.95), distinguishing it from the Yellow and Blue Samples (*p* < 0.05). Moreover, it was strongly associated with intense (2.92), indicating a more vivid and stimulating sensory profile. These findings are consistent with previous literature highlighting red as a color that enhances appetite and conveys positive emotional responses.

The Yellow Sample showed an intermediate profile. It did not significantly outperform the other colors in terms of hedonic or positive emotions; however, it was more closely associated with curious and refreshed, suggesting that yellow may elicit a sense of novelty and freshness.

In contrast, the Blue Sample was consistently linked to less favorable perceptions. It had the lowest mean scores for pleasant (3.66) and with pleasure (2.61) and was more frequently associated with negative descriptors such as nauseous and strange. Additionally, blue received higher values for no appetite, reinforcing the notion that this color is less congruent with food-related pleasure.

Overall, these findings confirm that food color significantly influences consumer emotions and hedonic perception, with red being the most positively perceived, yellow associated with curiosity and freshness, and blue eliciting less favorable responses. This reinforces the critical role of visual cues in multisensory food perception and highlights the importance of color as a design element in gastronomy and consumer science.

**Figure 1:**
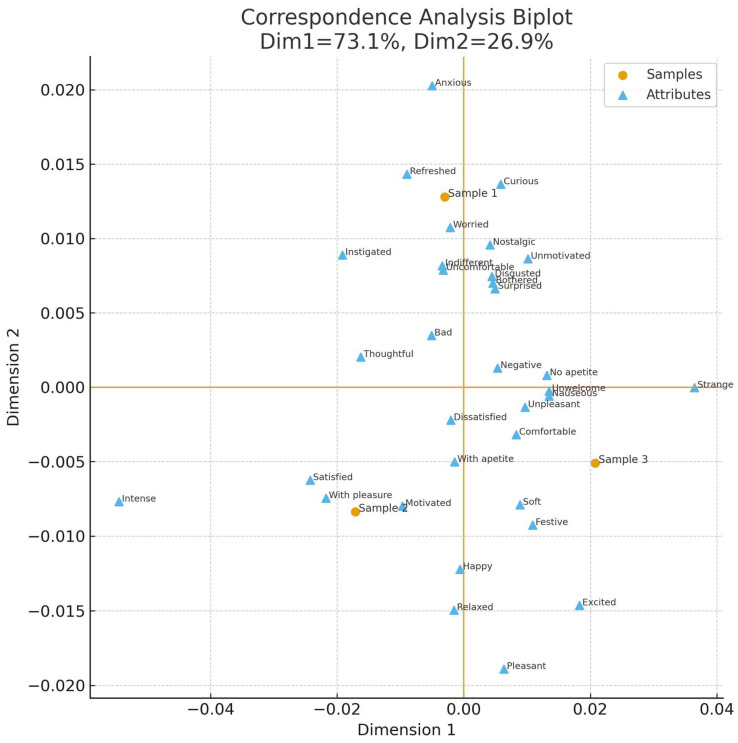
Correspondence Analysis (CA) biplot illustrating the relationship between samples (represented as circles) and RATA terms (represented as triangles). The proximity between terms and samples indicates stronger associations, while the distribution along the two dimensions explains the major sources of variance in consumer responses.

Correspondence Analysis was employed to explore the associations between samples and sensory terms. The first two dimensions together explained 68% of the total variance, with 42% for Dimension 1 and 26% for Dimension 2. The biplot (Figure 1) revealed groupings of samples around specific descriptors, allowing the identification of distinct sensory signatures. Significant attributes identified by Friedman’s test (*p* < 0.05) included pleasant, with pleasure, intense, strange, disgusted, indifferent, and relaxed, among others.

### 3.4. Acceptance Analysis

The hedonic evaluation revealed significant differences among the samples. The Red sample achieved the highest mean score (7.27), followed by the Blue sample (7.03) and the Yellow sample (6.82). Although the differences between Blue and Yellow were not significant, both scored lower than Red, confirming its stronger positive impact on consumer acceptance (Table 4).

**Figure 2:**
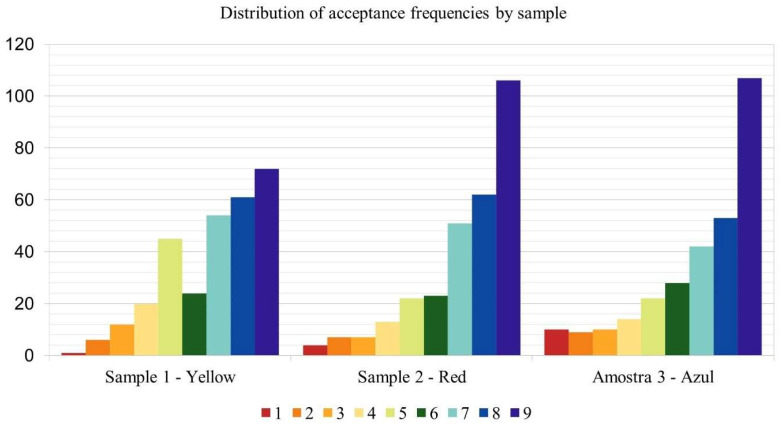
Distribution of acceptance frequencies by sample.

Figure 2 illustrates the frequency distribution of overall evaluation scores for each sample. The Red and Blue samples exhibited a higher frequency of positive evaluations (>7) compared to intermediate (>4 and <6) and negative evaluations (<3). The Yellow sample, although predominantly receiving positive evaluations, displayed a more balanced distribution across all levels, indicating greater variability in individual preferences. Notably, the Blue sample showed a higher incidence of negative evaluations than the other samples.

The evaluation of associations between emotional and sensory terms obtained through the RATA method and overall liking revealed consistent and statistically robust patterns. Pearson’s correlations (Table 5) indicated that attributes such as *pleasant* (r = 0.62, *p* < 0.001), *with pleasure* (r = 0.58, *p* < 0.001), and *satisfied* (r = 0.55, *p* < 0.001) showed the strongest positive correlations with overall acceptance, acting as important drivers of liking. Conversely, descriptors such as *disgusted* (r = −0.49, *p* < 0.001) and *negative* (r = −0.44, *p* < 0.001) showed significant negative correlations, characterizing rejection drivers.

**Figure 3:**
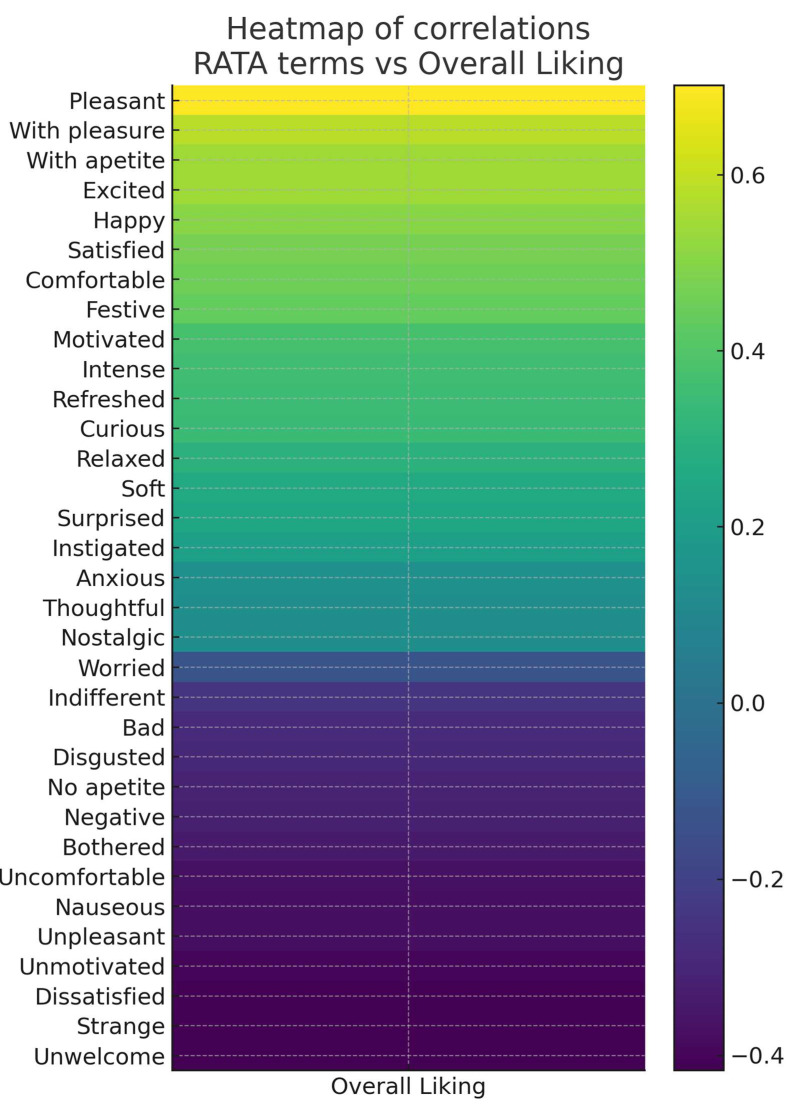
Heatmap of correlations between RATA terms and Overall Liking.

The correlation heatmap (Figure 3) provided an integrated visualization of these results, highlighting clusters of positive attributes associated with higher acceptance and, in contrast, clusters of terms related to rejection.

**Figure 4:**
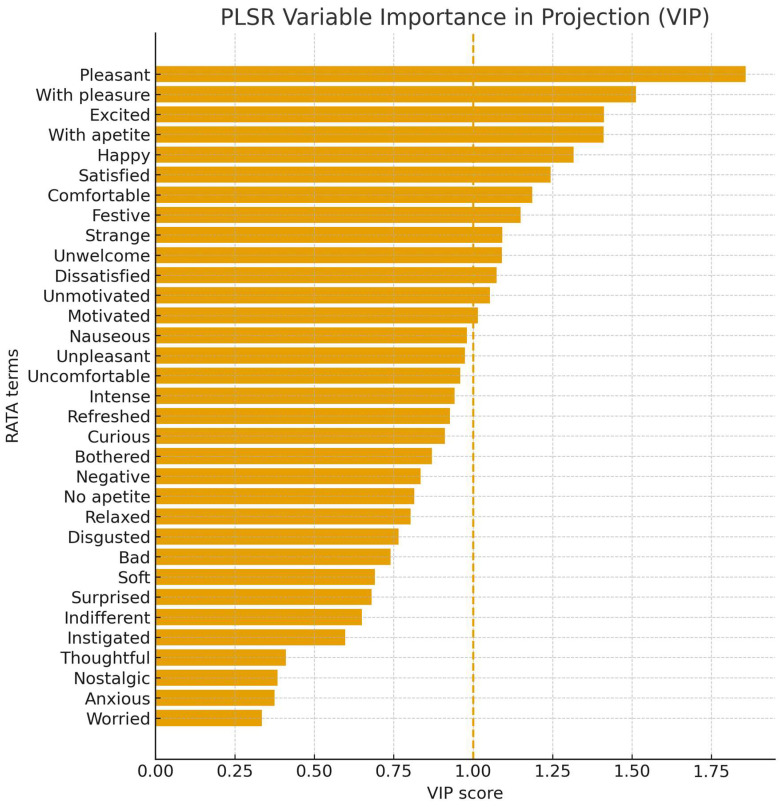
PLSR Variable Importance in Projection (VIP). (The dashed line indicates the threshold VIP = 1).

In the Partial Least Squares Regression (PLSR), a clear association was identified between the RATA terms and overall liking. The regression coefficients (Table 6) revealed that attributes such as *pleasant*, *with pleasure*, and *satisfied* contributed positively to acceptance, whereas *disgusted* and *negative* exerted negative effects. Furthermore, the analysis of VIP values (Figure 4) demonstrated that terms with VIP > 1 were the main predictors in the model, thereby reinforcing the relevance of the attributes previously highlighted in the correlation analysis.

**Figure 5:**
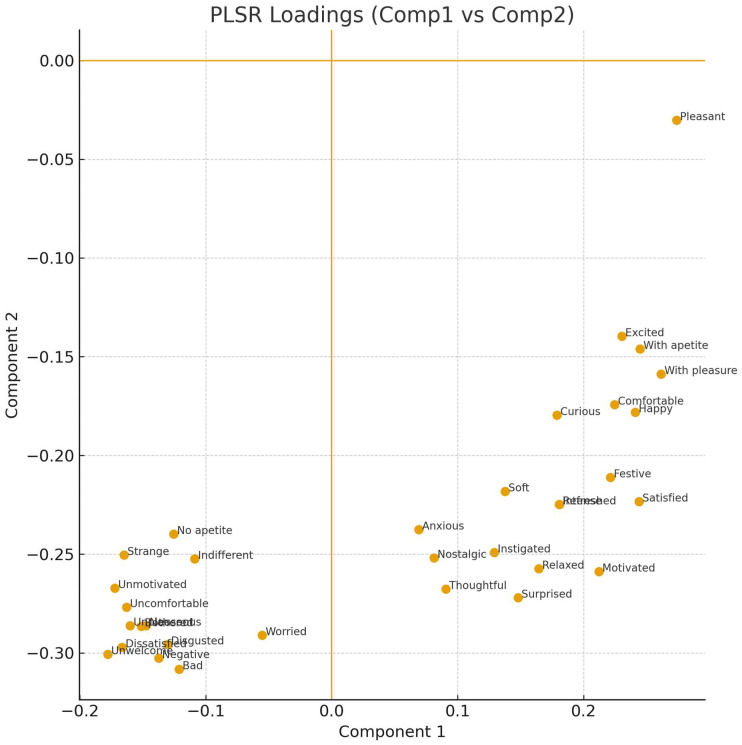
PLSR loadings (Component 1 vs. Component 2). (The plot shows the distribution of attributes, with positive descriptors grouped in the same direction as Overall Liking, in contrast to negative attributes).

The PLSR loadings biplot (Figure 5) highlighted the proximity of positive attributes, which clustered in the same direction as acceptance, in contrast to negative attributes.

Taken together, the results obtained from the combination of correlation analysis, CA, and PLSR reinforce the relevance of emotional and hedonic attributes as key determinants of product acceptance. The consistency across methods highlights the robustness of the conclusions, suggesting that future developments and formulation strategies should prioritize the intensification of positive descriptors (pleasant, with pleasure, satisfied) and the reduction in negative attributes (disgusted, negative) to maximize overall product acceptance.

### 3.5. Analysis of Influences

**Figure 6:**
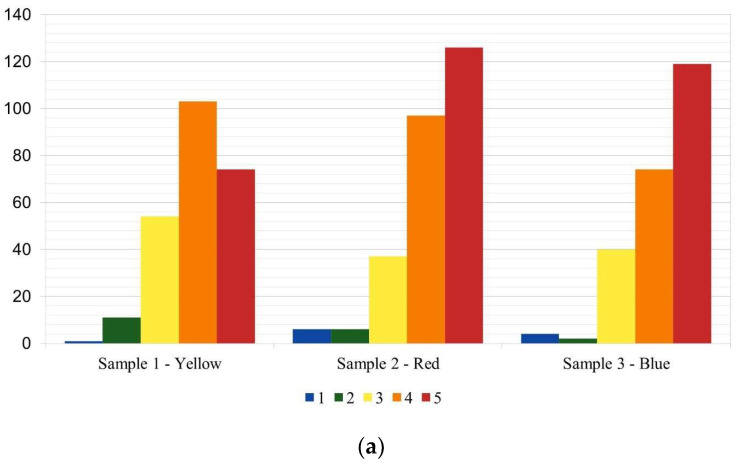

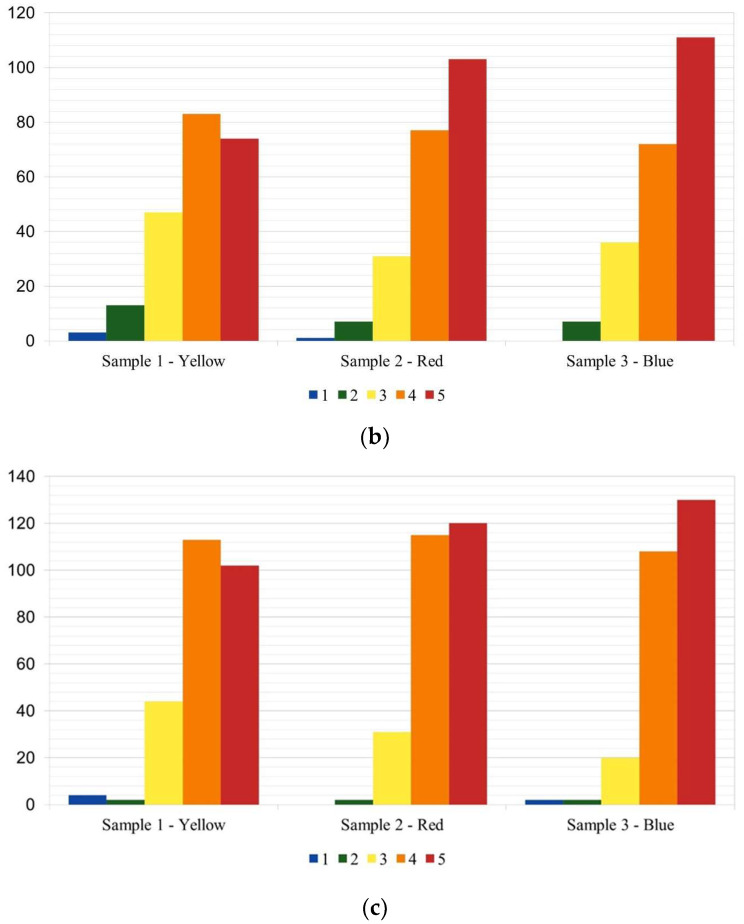
Frequency distributions of hedonic responses to plating-related questions: (**a**) Evaluation of whether participants liked the main color of the sample; (**b**) Perceived influence of plates on emotional responses; (**c**) Perceived influence of food shapes and arrangements on the emotions evaluated. Results are expressed as percentage frequencies across the 5-point hedonic scale for each sample (red, yellow, and blue).

For the question “Do you like the main color present in the sample?”, although the Red and Blue samples did not differ significantly (Table 7), their frequency distributions revealed distinct patterns (Figure 6a). The Red sample exhibited a predominance of very positive responses (≥4), whereas the Yellow sample showed a more even distribution across all response levels.

Regarding the influence of plates on emotional perception, Table 7 presents the results. Figure 6b indicates that the Red and Blue samples predominantly received very positive responses (≥4), while the Yellow sample displayed more balanced distributions, highlighting variability in participants’ perceptions.

For the final question, *“Did the shapes and arrangements of the elements influence the perception of the emotions evaluated?”*, Table 7 details the frequencies of positive responses. Figure 6c shows that the Red and Blue samples again exhibited a higher concentration of very positive responses. In contrast, the Yellow sample presented a notable number of intermediate responses alongside positive ones.

For the question regarding the influence of plates on the perception of emotions, a high percentage of positive responses was observed for all samples, indicating that, to some extent, the evaluation of emotions was affected by the plates used. According to Spence et al. [52], plate colors strongly influence people’s perception of food, extending beyond a decorative function to impact appetite and even the perceived flavor of the food.

The authors also state that the color of the plate can alter the perceived color of the food through a phenomenon called simultaneous color contrast. In this case, an object in the foreground appears to change in color depending on the background against which it is placed. For example, the yellow of scrambled eggs on a yellow plate may appear paler due to this contrast, affecting actual perception [52]. Therefore, further studies are needed to better understand how this color interaction influences emotional perception.

The other question assessed the influence of the shape and arrangement of elements on the plate. Again, a high percentage of positive responses was observed for all samples, indicating that the evaluation of emotions was directly influenced by food shape and arrangement. Food presentation on plates can positively affect consumer behavior; however, an imbalance in presentation may have negative effects, as people tend to prefer food arranged in an organized and aesthetically pleasing manner [43,53,54].

### 3.6. CATA Analysis of Basic Tastes and Oral Sensations

The CATA analysis of basic tastes and oral sensations was conducted using Cochran’s Q test, followed by paired multiple comparisons with McNemar’s test and Bonferroni correction (Table 8).

A significant difference was observed among the samples in terms of perceived tastes. The Red sample was primarily associated with the spicy attribute, the Yellow sample with sour, salty, and fatty, and the Blue sample with sweet.

According to Shermer and Levitan [55], red-colored foods are associated with thermal heat and spicy compounds, creating the perception of spiciness even when the product does not contain capsaicin or similar compounds. Red has also been linked to other attributes [51], but this association was distant from the positioning of the sample. Moreover, although red hues are generally associated with sweetness rather than bitterness, darker colors such as violet or purple have been repeatedly linked to bitterness in crossmodal taste–color correspondence studies [56]. In this light, the violet tone of beetroot in the Red sample may have influenced some participants to perceive a bitter sensation.

The Blue sample was associated with the sweet attribute, consistent with literature indicating that blue is often linked to sweet products such as cakes and candies [52]. Although blue can sometimes evoke saltiness [43], this effect was not observed in the present study. Thus, the presence of blue elements in this context suggests a perception of sweetness, even though the sample itself was not a dessert.

The Yellow sample was associated with the sour and salty attributes. Yellow is commonly linked to sourness on the palate [43], while the association with saltiness is less documented, suggesting that another color, such as white, which was visible in the sample, may have influenced this perception. Interestingly, yellow coloration has also been linked to perceptions of fattiness in certain foods. For example, a recent study on poultry meat quality reported that consumers associated yellowish tones in chicken with negative attributes such as rancid, unnatural, unfamiliar, and notably fatty, suggesting that yellow may evoke expectations of excess fat content or reduced freshness in some contexts [57]. This finding reinforces the notion that the interpretation of yellow in food products is context-dependent, capable of eliciting both positive associations (e.g., freshness, citrus-like acidity) and negative ones (e.g., fattiness, rancidity).

### 3.7. Limitations of the Study

This study has some limitations. Evaluations were conducted online using food photographs, which limited control over conditions such as lighting, screen resolution, and distractions, potentially influencing color perception and emotional responses. The sample consisted only of Brazilian consumers, reducing the generalizability of the findings across cultures. In addition, only three plating colors (red, yellow, and blue) were tested, restricting the scope of the conclusions. Although the sample size (n = 295) was adequate, no formal power analysis was performed, which should be considered when interpreting the results.

## 4. Conclusions

The colors used in plating proved fundamental to the perception of emotions, reinforcing color as a relevant sensory attribute in gastronomy and a determinant of consumer acceptance. The findings align with existing literature for red and yellow, whose symbolic meanings were consistent across food and non-food contexts. In contrast, blue revealed a notable divergence between its conventional associations in other domains and its sensory interpretation in food, underscoring the need for deeper exploration of atypical hues in gastronomy.

Associations between colors and basic tastes followed established patterns, except for blue, which once again showed distinct differences, highlighting its unique role in shaping perception. Beyond the effect of color itself, contextual elements such as plate design and food arrangement also influenced perception, demonstrating the multisensory nature of food experiences. Moreover, the use of RATA proved effective in identifying the sensory–emotional dimensions most closely related to acceptance, offering practical guidance for product development.

Taken together, these findings demonstrate that plating color is not only an aesthetic factor but also a determinant of emotional response and hedonic acceptance, reinforcing the role of multisensory cues in gastronomy. Future studies should expand the range of tested colors, explore their combinations, and include cross-cultural evaluations to strengthen external validity and broaden the applicability of the results.

Despite the promising results, this study has limitations. The use of photographic stimuli and a sample restricted to Brazilian consumers limits ecological and cross-cultural generalization. Future research should replicate these findings in face-to-face settings, with multisensory stimuli and more diverse populations, to validate and extend the results.

Practically, these findings provide valuable insights for chefs, product developers, and sensory designers seeking to integrate color as a strategic element in dish presentation and product formulation. Applying color–emotion correspondences in gastronomic design may enhance consumers’ sensory engagement, emotional connection, and overall dining experience.

## Figures and Tables

**Table 1:** Selection of samples according to their color.

Sample	Color	Plating	Composition
Sample 1	Yellow	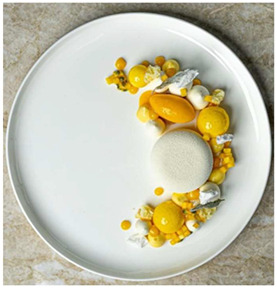 Chef’s Roll, Inc. [21]	Cream cheese mousse, vanilla whipped ganache, passion fruit cremeux, meringue, lemon madeleine sponge, passion fruit and mango ice cream, and banana cremeux.
Sample 2	Red	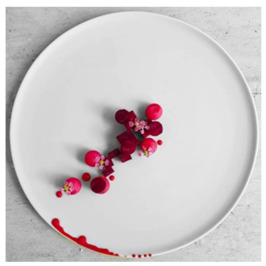 Chef Sijo Chandran [22]	Mini beetroot, truffled honey, goat cheese, and beetroot puree.
Sample 3	Blue	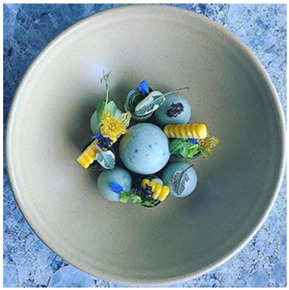 Mustafa Yankavi [23]	Blue cheese, inkfish sponge cake, coconut milk foam, cucumber flower, blue borage flower, micro pea shoots, and sage

**Table 2:** Multiple comparisons between CATA test results for each attribute in all samples using the McNemar procedure.

Terms	Cochran’s Q (*p*-Values)	Sample 1 (Yellow)	Sample 2 (Red)	Sample 3 (Blue)
Anxious	<0.0001	0.790 ^b^	0.725 ^a^	0.705 ^a^
Bad	0.000	0.732 ^b^	0.698 ^a^	0.675 ^a^
Bothered	0.005	0.763 ^b^	0.715 ^a^	0.708 ^a^
Comfortable	0.101	0.854 ^a^	0.817 ^a^	0.814 ^a^
Curious	<0.0001	0.922 ^b^	0.851 ^a^	0.847 ^a^
Disgusted	0.045	0.756 ^b^	0.715 ^a^	0.722 ^ab^
Dissatisfied	0.011	0.739 ^b^	0.712 ^ab^	0.692 ^a^
Excited	0.484	0.868 ^a^	0.844 ^a^	0.858 ^a^
Festive	0.292	0.831 ^a^	0.803 ^a^	0.803 ^a^
Happy	0.184	0.847 ^a^	0.834 ^a^	0.810 ^a^
Indifferent	0.002	0.763 ^b^	0.719 ^ab^	0.698 ^a^
Instigated	<0.0001	0.817 ^b^	0.780 ^b^	0.729 ^a^
Intense	<0.0001	0.810 ^b^	0.827 ^b^	0.705 ^a^
Motivated	0.016	0.783 ^b^	0.769 ^ab^	0.732 ^a^
Nauseous	0.001	0.753 ^b^	0.705 ^a^	0.698 ^a^
Negative	0.013	0.736 ^b^	0.698 ^ab^	0.692 ^a^
No appetite	0.053	0.780 ^b^	0.736 ^a^	0.742 ^ab^
Nostalgic	0.000	0.756 ^b^	0.705 ^a^	0.698 ^a^
Pleasant	0.417	0.953 ^a^	0.946 ^a^	0.932 ^a^
Refreshed	0.000	0.824 ^b^	0.769 ^a^	0.739 ^a^
Relaxed	0.147	0.756 ^a^	0.749 ^a^	0.725 ^a^
Satisfied	0.000	0.800 ^b^	0.793 ^b^	0.729 ^a^
Soft	0.246	0.800 ^a^	0.773 ^a^	0.769 ^a^
Strange	0.008	0.810 ^b^	0.749 ^a^	0.800 ^ab^
Surprised	0.003	0.824 ^b^	0.773 ^a^	0.766 ^a^
Thoughtful	0.001	0.810 ^b^	0.783 ^ab^	0.736 ^a^
Uncomfortable	0.004	0.769 ^b^	0.725 ^a^	0.705 ^a^
Unmotivated	0.003	0.769 ^b^	0.715 ^a^	0.719 ^a^
Unpleasant	0.060	0.763 ^a^	0.725 ^a^	0.725 ^a^
Unwelcome	0.035	0.746 ^b^	0.705 ^a^	0.712 ^ab^
With appetite	0.039	0.871 ^a^	0.844 ^a^	0.820 ^a^
With pleasure	0.001	0.854 ^b^	0.847 ^b^	0.783 ^a^
Worried	<0.0001	0.739 ^b^	0.692 ^a^	0.675 ^a^

Values are expressed as mean ± standard deviation. Different letters within the same row indicate significant differences between samples according to the Bonferroni post hoc test (*p* < 0.05). The Cochran’s Q test was used to assess overall differences among samples.

**Table 3:** Mean RATA scores for each term evaluated across samples.

Term	Sample 1 (Yellow)	Sample 2 (Red)	Sample 3 (Blue)	Friedman (*p*-Value)
Pleasant	3.68 ± 1.37 ^a^	3.90 ± 1.35 ^a^	3.66 ± 1.54 ^a^	0.0229
Excited	3.18 ± 1.66 ^a^	3.23 ± 1.74 ^a^	3.11 ± 1.76 ^a^	0.6116
Anxious	1.78 ± 1.48 ^a^	1.69 ± 1.53 ^a^	1.59 ± 1.58 ^a^	0.0082
With appetite	2.65 ± 1.58 ^a^	2.66 ± 1.66 ^a^	2.49 ± 1.76 ^a^	0.0214
With pleasure	2.89 ± 1.67 ^a^	2.95 ± 1.70 ^a^	2.61 ± 1.86 ^a^	0.0056
Nauseous	1.19 ± 1.14 ^a^	1.01 ± 0.97 ^a^	1.14 ± 1.16 ^a^	0.0283
Comfortable	3.03 ± 1.64 ^a^	2.87 ± 1.75 ^a^	2.93 ± 1.84 ^a^	0.1775
Curious	3.62 ± 1.54 ^a^	3.39 ± 1.75 ^a^	3.36 ± 1.84 ^a^	0.0610
Unpleasant	1.13 ± 1.03 ^a^	1.07 ± 1.06 ^a^	1.09 ± 1.07 ^a^	0.3271
Uncomfortable	1.14 ± 1.05 ^a^	1.08 ± 1.08 ^a^	1.14 ± 1.17 ^a^	0.3515
Unmotivated	1.26 ± 1.20 ^a^	1.09 ± 1.15 ^a^	1.25 ± 1.27 ^a^	0.0045
Disgusted	1.14 ± 1.10 ^a^	0.97 ± 0.97 ^a^	1.03 ± 1.07 ^a^	0.0026
Strange	1.71 ± 1.43 ^a^	1.43 ± 1.36 ^b^	1.73 ± 1.50 ^a^	0.0003
Happy	3.05 ± 1.70 ^a^	3.03 ± 1.72 ^a^	2.84 ± 1.80 ^a^	0.0152
Festive	2.90 ± 1.79 ^a^	2.85 ± 1.85 ^a^	2.72 ± 1.83 ^a^	0.1697
Bothered	1.25 ± 1.19 ^a^	1.10 ± 1.10 ^a^	1.13 ± 1.12 ^a^	0.0340
Unwelcome	1.19 ± 1.18 ^a^	1.09 ± 1.14 ^a^	1.15 ± 1.22 ^a^	0.1204
Indifferent	1.33 ± 1.16 ^a^	1.11 ± 1.07 ^b^	1.16 ± 1.17 ^a^	0.0014
Dissatisfied	1.19 ± 1.18 ^a^	1.15 ± 1.19 ^a^	1.19 ± 1.27 ^a^	0.4148
Instigated	2.51 ± 1.71 ^a^	2.30 ± 1.79 ^a^	2.11 ± 1.82 ^b^	0.0007
Intense	2.56 ± 1.78 ^a^	2.92 ± 1.87 ^b^	2.01 ± 1.77 ^c^	0.0000
Bad	1.01 ± 0.90 ^a^	1.00 ± 1.01 ^a^	0.94 ± 0.97 ^a^	0.0112
Motivated	2.49 ± 1.78 ^a^	2.50 ± 1.78 ^a^	2.18 ± 1.82 ^a^	0.0034
Negative	1.07 ± 1.03 ^a^	0.99 ± 0.98 ^a^	1.11 ± 1.16 ^a^	0.1371
Nostalgic	1.41 ± 1.32 ^a^	1.25 ± 1.32 ^a^	1.49 ± 1.53 ^a^	0.0305
Thoughtful	2.27 ± 1.65 ^a^	2.17 ± 1.69 ^a^	2.25 ± 1.80 ^a^	0.5480
Worried	1.05 ± 0.99 ^a^	0.96 ± 0.96 ^a^	1.03 ± 1.10 ^a^	0.0928
Refreshed	2.43 ± 1.69 ^a^	2.14 ± 1.67 ^a^	2.24 ± 1.79 ^a^	0.0667
Relaxed	2.07 ± 1.65 ^a^	1.96 ± 1.64 ^a^	2.24 ± 1.84 ^a^	0.0331
Satisfied	2.59 ± 1.75 ^a^	2.62 ± 1.78 ^a^	2.39 ± 1.90 ^a^	0.2062
No appetite	1.59 ± 1.41 ^a^	1.34 ± 1.29 ^a^	1.50 ± 1.41 ^a^	0.0483
Soft	2.45 ± 1.67 ^a^	2.15 ± 1.69 ^a^	2.49 ± 1.84 ^a^	0.0296
Surprised	2.64 ± 1.72 ^a^	2.50 ± 1.83 ^a^	2.62 ± 1.86 ^a^	0.6313

Values are expressed as mean ± standard deviation. Different letters within the same row indicate significant differences between samples according to the Bonferroni post hoc test (*p* < 0.05). The Friedman test was used to assess overall differences among samples.

**Table 4:** Mean hedonic acceptance scores (±standard error and 95% CI) for each sample.

Sample	Means	Standard Error	Lower Limit (95%)	Upper Limit (95%)
Sample 1 (Yellow)	6.820 ^b^	0.119	6.587	7.054
Sample 2 (Red)	7.275 ^a^	0.119	7.041	7.508
Sample 3 (Blue)	7.027 ^ab^	0.119	6.793	7.261

Different letters indicate significant differences by Tukey’s test, *p* < 0.05.

**Table 5:** Pearson correlations between RATA terms and Overall Liking.

Term	Pearson R	*p*-Value
Pleasant	0.702	0.0
With pleasure	0.581	0.0
With appetite	0.542	0.0
Excited	0.540	0.0
Happy	0.505	0.0
Satisfied	0.472	0.0
Comfortable	0.453	0.0
Festive	0.438	0.0
Unwelcome	−0.417	0.0
Strange	−0.414	0.0
Dissatisfied	−0.409	0.0
Unmotivated	−0.403	0.0
Motivated	0.375	0.0
Unpleasant	−0.373	0.0
Nauseous	−0.372	0.0
Uncomfortable	−0.368	0.0
Intense	0.359	0.0
Refreshed	0.352	0.0
Curious	0.346	0.0
Bothered	−0.334	0.0
Negative	−0.320	0.0
No appetite	−0.310	0.0
Relaxed	0.300	0.0
Disgusted	−0.293	0.0
Bad	−0.283	0.0
Soft	0.261	0.0
Indifferent	−0.249	0.0
Surprised	0.242	0.0
Instigated	0.214	0.0
Anxious	0.144	0.0
Thoughtful	0.142	0.0
Nostalgic	0.141	0.0
Worried	−0.128	0.0001

**Table 6:** PLSR coefficients and Variable Importance in Projection (VIP) values.

Term	Coefficient	VIP
Pleasant	0.222	1.857
With pleasure	0.155	1.513
Excited	0.155	1.412
With appetite	0.144	1.411
Happy	0.123	1.316
Satisfied	0.100	1.244
Comfortable	0.104	1.187
Festive	0.097	1.149
Strange	−0.128	1.092
Unwelcome	−0.120	1.090
Dissatisfied	−0.124	1.074
Unmotivated	−0.115	1.053
Motivated	0.064	1.016
Nauseous	−0.116	0.981
Unpleasant	−0.106	0.974
Uncomfortable	−0.100	0.959
Intense	0.080	0.941
Refreshed	0.075	0.927
Curious	0.073	0.911
Bothered	−0.089	0.871
Negative	−0.091	0.835
No appetite	−0.094	0.815
Relaxed	0.056	0.803
Disgusted	−0.080	0.765
Bad	−0.081	0.741
Soft	0.053	0.690
Surprised	0.032	0.681
Indifferent	−0.069	0.650
Instigated	0.030	0.597
Thoughtful	0.016	0.411
Nostalgic	0.023	0.385
Anxious	0.035	0.375
Worried	−0.036	0.335

Note: Coefficients indicate the direction of association with Overall Liking. Terms with VIP > 1 were considered relevant predictors.

**Table 7:** Mean values (±SE) for the three evaluated questions, assessed using the 5-point hedonic scale.

Question/Sample	Sample 1 (Yellow)	Sample 2 (Red)	Sample 3 (Blue)
*Do you like the main color present in the sample?*	3.979 ^b^ ± 0.057	4.217 ^a^ ± 0.054	4.264 ^a^ ± 0.058
*Did the plates used influence emotional perception?*	3.964 ^b^ ± 0.059	4.251 ^a^ ± 0.060	4.270 ^a^ ± 0.059
*Did the shapes and arrangements influence the perception of emotions?*	4.158 ^b^ ± 0.046	4.317 ^a^ ± 0.046	4.382 ^a^ ± 0.047

Note: Values are expressed as mean ± standard error. Different superscript letters in the same row indicate significant differences among samples according to the applied post hoc test (*p* < 0.05).

**Table 8:** Multiple comparisons between the results of the CATA test for basic tastes and sensations in all samples using the McNemar (Bonferroni) procedure.

Attributes	Cochran’s Q (*p*-Values)	Sample 1 (Yellow)	Sample 2 (Red)	Sample 3 (Blue)
Sweet	<0.0001	0.556 ^a^	0.603^a^	0.776 ^b^
Salty	<0.0001	0.349 ^b^	0.244^ab^	0.180 ^a^
Bitter	0.0000	0.078 ^a^	0.193^b^	0.129 ^ab^
Sour	<0.0001	0.525 ^c^	0.315^b^	0.142 ^a^
Umami	0.6070	0.122 ^a^	0.132^a^	0.105 ^a^
Fatty	<0.0001	0.397 ^c^	0.027^a^	0.224 ^b^
Astringent	0.6530	0.186 ^a^	0.207^a^	0.176 ^a^
Spicy	<0.0001	0.058 ^a^	0.298^b^	0.068 ^a^

Note: Different letters indicate significant differences according to the applied statistical tests (*p* < 0.05).

## Data Availability

The data presented in this study are available on request from the corresponding author. The data are not publicly available due to privacy restrictions.

## References

[1] Neri-Numa I.A., Carvalho-Silva L.B., Morales J.P., Pastore G.M. (2017). Genipin: A natural blue pigment for food and health purposes. Trends Food Sci. Technol..

[2] Grzybowski A., Kupidura-Majewski K. (2019). What is color and how it is perceived?. Clin. Dermatol..

[3] Yue L., Castillo J., Gonzalez A.C., Neitz J., Humayun M.S. (2021). Restoring color perception to the blind. Ophthalmology.

[4] O’Connor Z. (2021). Traditional colour theory: A review. Color Res. Appl..

[5] Michel C., Velasco C., Gatti E., Spence C. (2015). Rotating plates: Online study demonstrates the importance of orientation in the plating of food. Food Qual. Prefer..

[6] Schifferstein H.N., Kudrowitz B.M., Breuer C. (2020). Food perception and aesthetics—Linking sensory science to culinary practice. J. Culin. Sci. Technol..

[7] Paakki M., Sandell M., Hopia A. (2019). Visual attractiveness depends on colorfulness and color contrasts in mixed salads. Food Qual. Prefer..

[8] Schifferstein H.N.J., Wehrle T., Carbon C.-C. (2019). Consumer Expectations for Vegetables with Typical and Atypical Colors: The Case of Carrots. Food Qual. Prefer..

[9] Ismael D., Ploeger A. (2019). Development of a sensory method to detect food-elicited emotions using emotion–color association and eye-tracking. Foods.

[10] Fugate J.M.B., Franco C.L. (2019). What color is your anger? Assessing color–emotion pairings in English speakers. Front. Psychol..

[11] Lin Y., Yang Y., Guo J., Luo M.R. (2023). Advances in color science: From color perception to color metrics and its applications in illuminated environments. Front. Psychol..

[12] Velasco C., Barbosa Escobar F., Spence C., Olier J.S. (2023). The Taste of Colours. Food Qual. Prefer..

[13] Steiner K., Florack A. (2023). The Influence of Packaging Color on Consumer Perceptions of Healthfulness: A Systematic Review and Theoretical Framework. Foods.

[14] Nagy L.B., Temesi Á. (2024). Color Matters: A Study Exploring the Influence of Packaging Colors on University Students’ Perceptions and Willingness to Pay for Organic Pasta. Foods.

[15] Szmagara A. (2024). Blue in Food and Beverages—A Review of Socio-Cultural, Economic, and Environmental Implications. Sustainability.

[16] Schlintl C., Schienle A. (2020). Effects of Coloring Food Images on the Propensity to Eat: A Placebo Approach With Color Suggestions. Front. Psychol..

[17] Spence C. (2021). What’s the Story With Blue Steak? On the Unexpected Popularity of Blue Foods. Front. Psychol..

[18] Michel C., Velasco C., Gatti E., Spence C. (2014). A taste of Kandinsky: Assessing the influence of the artistic visual presentation of food on the dining experience. Flavour.

[19] Cosme F., Rocha T., Marques C., Barroso J., Vilela A. (2025). Innovative Approaches in Sensory Food Science: From Digital Tools to Virtual Reality. Appl. Sci..

[20] Jo D.-M., Han S.-J., Ko S.-C., Kim K.W., Yang D., Kim J.-Y., Oh G.-W., Choi G., Lee D.-S., Tabassum N. (2025). Application of Artificial Intelligence in the Advancement of Sensory Evaluation of Food Products. Trends Food Sci. Technol..

[21] Chef’s Roll, Inc Exotic Cheesecake. *Instagram Post*, 18 March 2020. https://www.instagram.com/p/B94YSs2ghX4/.

[22] Chef Sijo Chandran Baby Beets. *Instagram Post*, 17 May 2021. https://www.instagram.com/p/CO9uXICFRAE/.

[23] Mustafa Yankayi Blue Cheese. *Instagram Post*, 9 July 2017. https://www.instagram.com/p/BWUxvSMHjaS/.

[24] King S.C., Meiselman H.L. (2010). Development of a method to measure consumer emotions associated with foods. Food Qual. Prefer..

[25] Souza C.M., Pinto G.A., Barros T.F., Garruti D.S., Sousa P.H.M. (2022). Development of the coffee taster’s emotion wheel for the coffee drinking experience. Int. J. Gastron. Food Sci..

[26] Chaya C., Pacoud J., Ng M., Fenton A., Hort J. (2015). Developing a reduced consumer-led lexicon to measure emotional response to beer. Food Qual. Prefer..

[27] Minim V.P.R. (2018). Análise Sensorial: Estudos Com Consumidores.

[28] Almujlli G., Alrabah R., Al-Ghosen A., Munshi F. (2022). Conducting virtual focus groups during the COVID-19 epidemic utilizing videoconferencing technology: A feasibility study. Cureus.

[29] Maciel J.B., de Oliveira Silva Y., Santos S.S., Dionísio A.P., Machado de Sousa P.H., Garruti D.S. (2022). Plant-based gastronomic products based on freeze-dried cashew fiber. Int. J. Gastron. Food Sci..

[30] Williamson D.A., Allen H.R., Martin P.D., Alfonso A.J., Gerald B., Hunt A. (2003). Comparison of Digital Photography to Weighed and Visual Estimation of Portion Sizes. J. Am. Diet. Assoc..

[31] Jonauskaite D., Abdel-Khalek A.M., Abu-Akel A., Al-Rasheed A.S., Antonietti J.-P., Ásgeirsson Á.G., Atitsogbe K.A., Barma M., Barratt D., Bogushevskaya V. (2019). The sun is no fun without rain: Physical environments affect how we feel about yellow across 55 countries. J. Environ. Psychol..

[32] Labrecque L.I., Milne G.R. (2011). Exciting red and competent blue: The importance of color in marketing. J. Acad. Mark. Sci..

[33] Bálizs B. (2021). Meanings of the color yellow and its color associates, yellow-black and yellow-green. Hung. Cult. Stud..

[34] Fikrlova J., Martončik M., Adamkovič M., Kačmár P. (2019). The power of red: The influence of colour on evaluation and failure—A replication. Acta Psychol..

[35] Gnambs T., Appel M., Kaspar K. (2015). The effect of the color red on encoding and retrieval of declarative knowledge. Learn. Individ. Differ..

[36] Tanaka A., Tokuno Y. (2011). The effect of the color red on avoidance motivation. Soc. Behav. Pers..

[37] Lunardo R., Saintives C., Chaney D. (2021). Food packaging and the color red: How negative cognitive associations influence feelings of guilt. J. Bus. Res..

[38] Mehta R., Zhu R. (2009). Blue or red? Exploring the effect of color on cognitive task performances. Science.

[39] Martinez L.M., Rando B., Agante L., Abreu A.M. (2021). True colors: Consumers’ packaging choices depend on the color of retail environment. J. Retail. Consum. Serv..

[40] Neves M.I.L., Fernandes A.S., Santos J.C., Sousa P.H.M., Garruti D.S. (2021). Natural blue food colorants: Consumer acceptance, current alternatives, trends, challenges, and future strategies. Trends Food Sci. Technol..

[41] Alley R.L., Alley T.R. (1998). The Influence of Physical State and Color on Perceived Sweetness. J. Psychol. Interdiscip. Appl..

[42] Velasco C., Veflen N. (2021). Aesthetic Plating and Motivation in Context. Int. J. Gastron. Food Sci..

[43] Watson L. (1971). The Omnivorous Ape.

[44] Spence C. (2018). What Is So Unappealing about Blue Food and Drink?. Int. J. Gastron. Food Sci..

[45] Suzuki M., Imai H., Nakae S., Yoshida K., Yamaguchi H. (2017). Color of Hot Soup Modulates Postprandial Satiety, Thermal Sensation, and Body Temperature in Young Women. Appetite.

[46] Spence C. (2019). On the Relationship(s) Between Color and Taste/Flavor. Psychol. Res..

[47] Palmer S.E., Schloss K.B. (2010). An Ecological Valence Theory of Human Color Preference. Proc. Natl. Acad. Sci. USA.

[48] Shankar M.U., Levitan C., Spence C. (2010). Grape Expectations: The Role of Cognitive Influences in Color–Flavor Interactions. Conscious. Cogn..

[49] Marinetti F.T., Brill S. (2014). The Futurist Cookbook.

[50] Hitchcock A., Gottlieb S. (2003). Alfred Hitchcock: Interviews.

[51] Spence C., Wan X., Woods A. (2015). On tasty colours and colourful tastes? Assessing, explaining, and utilizing crossmodal correspondences between colours and basic tastes. Flavour.

[52] Spence C., Michel C., Deroy O. (2014). Plating manifesto (II): The art and science of plating. Flavour.

[53] Zellner D.A., Loss C.R., Zearfoss J., Remolina S. (2011). Neatness counts: How plating affects liking for the taste of food. Appetite.

[54] Velasco C., Woods A.T., Petit O., Cheok A.D., Spence C. (2016). On the importance of balance to aesthetic plating. Int. J. Gastron. Food Sci..

[55] Shermer D.Z., Levitan C.A. (2014). Red hot: The crossmodal effect of color intensity on perceived piquancy. Multisens. Res..

[56] Spence C., Levitan C.A. (2021). Explaining crossmodal correspondences between colours and tastes. i-Perception.

[57] Kennedy O.B., Stewart-Knox B.J., Mitchell P.C., Thurnham D.I. (2005). Flesh colour dominates consumer preference for chicken. Appetite.

